# The Evaluation of Daily Life Activities after Application of an Osseointegrated Prosthesis Fixation in a Bilateral Transfemoral Amputee: A Case Study

**DOI:** 10.1097/MD.0000000000001416

**Published:** 2015-09-11

**Authors:** Stephanie A.F. Schalk, Niels Jonkergouw, Fred van der Meer, Willem M. Swaan, Horst-H. Aschoff, Peter van der Wurff

**Affiliations:** From the Military Rehabilitation Centre Aardenburg, Korte Molenweg 3, 3941 PW, Doorn, The Netherlands (SAFS, NJ, FVDM, WMS, HHA, PVDW); Sana Krankenhaus Süd Klinik für Plastische, Hand- und Rekonstruktive Chirurgie Kronsforder Allee 71–73, 23560 Lübeck, Schleswig-Holstein, Germany (HHA); and Department of Physical Therapy, HU University of Applied Sciences Utrecht, Bolognalaan 101, 3584 CJ, Utrecht, The Netherlands (PVDW).

## Abstract

Individuals with a transfemoral amputation (TFA) may experience limitations in daily life due to reduced mobility and prosthesis-related problems. An osseointegrated prosthesis fixation (OPF) procedure in amputees might contribute to a solution for patients with short stumps or socket-related problems. To date, no study has specifically described the application of an OPF procedure in individuals with a TFA. This study evaluated the level of daily life activities of a 21-year old service member with a bilateral TFA and cerebral trauma. Due to a short stump length and coordination problems, an OPF procedure was deemed the most suitable option.

The result of this procedure and the rehabilitation program showed an increased mobility and satisfaction as obtained by the assessment of life habits questionnaire (LIFE-H) and lower extremity functional scale. The participant was able to walk short distances and the Genium knee provided a stance position. Stair ambulation is impossible because of inadequate muscle capacity.

In this specific case we conclude that the quality of life improved through the use of an OPF. However, OPF might not be the appropriate device for every individual with TFA, due to varying bone compositions, co-morbidities, and limited clinical experience and unknown long-term effects.

## INTRODUCTION

Individuals with a transfemoral amputation (TFA) may experience limitations in daily life due to reduced mobility and prosthesis-related problems.^1–6^ Skin irritation, heat, and sweating in the prosthetic socket are frequently reported and appear to be the main factors that influence quality of life in transfemoral amputees.^7^ In patients with short stumps an additional challenge is encountered, ensuring that patients have sufficient socket control.

The introduction of an osseointegrated prosthesis fixation (OPF) procedure, which involves direct anchorage of an implant to the femur bone and integration with osseous tissue, contributes to reducing socket-related problems and also aims to enhance mobility.^8^ Several studies reported a better quality of life 2 years after receiving an OPF.^2,9–11^

A select number of studies mention individuals with both a bilateral TFA and an OPF; however, targeted data is lacking.^2,9–11^ Moreover, these studies do not describe the rehabilitation process in individuals with bilateral TFA and an OPF.

The aim of this case report is to evaluate the level of daily life activities of a patient with a bilateral TFA, before and after the application of an OPF procedure and auto-adaptive prosthetic knees.

## CASE DESCRIPTION

At 21 years of age, the patient was injured by an improvised explosive device (IED) during a military mission (June 2009). In this life-threatening situation, both legs were seriously injured, including multiple fractures, soft tissue, and vascular damage.^4^ Consequently, a bilateral TFA at the field hospital was inevitable. Furthermore, the patient was diagnosed with a traumatic cerebral contusion and a fractured mandible. The patient suffered from cognitive disabilities, experiencing a fragile and diminished memory and a reduced balance. The patient was repatriated to the Central Military Hospital (CMH) in Utrecht, and subsequently to the Military Rehabilitation Centre Aardenburg (MRC) in Doorn, the Netherlands.

After 5 months of neurological rehabilitation at the MRC, the patient achieved an acceptable outcome. The rehabilitation process was continued, focusing on the new aim of our patient, walking and standing in an upright position, and communicating with people at eye level. Initially, a socket-prosthesis seemed to be the logical first choice for the patient.

## PROSTHETIC PROCESS

**FIGURE 1:**
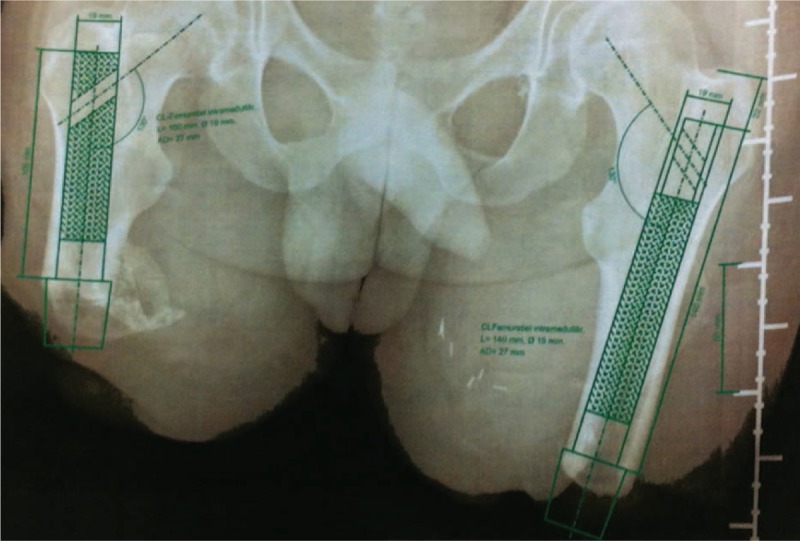
X-ray of participant stumps with computerized preparation for the osseointergration procedure.

The short stumps, with a length of 13 cm and 19 cm of the right and left leg, respectively, from the top of the femoral head to the end of the femur (Figure 1) were initially (January 2010) fitted with quadrilateral sockets to allow balance and gait training between parallel bars. Unfortunately, the patient had insufficient control of the socket while walking with a walking aid. As such, the prosthetic design was switched to an ischial containment fitting, instead of the previous quadrilateral, to improve gait stability. The stability improved, however, fitting problems persisted, with air inside the liner and rotation of the socket, leading to an increased risk of falling and a diminished level of daily life activities.

A bilateral above-knee suspension belt improved the above-mentioned aspects and directly increased functional use of the prosthesis. Unfortunately, after falling, the patient was unable to stand up. In an attempt to stand up, stump socket rotations are present, allowing air inside the liner. As a result, the patient had to intermittently doing on his prosthetics due to discomfort. Several attempts to control the problems related to rotation and fixation of the socket failed.

## OSSEOINTEGRATION

Despite the disappointing experience with the socket-prosthesis, the patient was highly motivated to achieve the above-mentioned aim, resulting in an OPF procedure as an option for the patient. The direct anchorage of an implant would solve the patients’ problem of inadequate prosthetic fit. Furthermore, the patient had no contraindications for the OPF procedure, such as diabetic and vascular complications, which would increase the risk of delayed wound healing and infection. In May 2012, 35 months after the initial accident, the patient underwent surgery for the OPF in the Sana Krankenhaus in Lübeck, Germany. Due to the short stump length, custom-made implants were required (Figure 1).

During the first stage of surgery, a pure titanium implant was fixated inside the medullar cavity of the femur.^9,12^ The post-operative recovery lasted 8 days. The second surgery was scheduled 6 weeks after the first surgery in order to provide adequate rest to enhance the healing process. During the second surgery, a titanium rod was inserted into the distal end of the implant and passed through the skin.^10^ The surgical procedures and postoperative recovery were successful, without complications.

## REHABILITATION

A week after the final surgery, rehabilitation treatment was initiated with the aim to improve weight bearing with respect to the osseointegrated implant. After approximately 4 weeks, full body weight bearing was achieved. The rehabilitation treatment consisted of balance training, muscle strength exercising, stability training of the hip and trunk and gait training in different floor conditions. Balance was trained via several dual-task exercises, using unstable surfaces in a computer-assisted rehabilitation environment (CAREN).^13^ Balance in daily life was improved through the use of assistive devices and a rolling walker.

During the rehabilitation treatment, the length of the prostheses was raised stepwise, from an initial length of 10 cm below the implant, by steps of 10 cm every other week, to a final length of 40 cm. Three months after the second surgery the maximal prosthetic length was achieved, after which, the prosthesis was provided with locking knees (3R41, Otto Bock), feet (1D10, Otto Bock), and shoes. At maximal length, the patient encountered right hip pain, a reaction to overuse of the hip, most likely related to the motivation of the patient. Accordingly, the rehabilitation staff advised the patient to temporarily decrease daily activities, resulting in immediate relief of the pain.

Five months after the second surgery, the patient finished the initial rehabilitation program. After a 2-month hiatus, a new rehabilitation program was initiated with the aim to walk while using auto-adaptive prosthetic knee joints in combination with 2 dynamic response feet (1C63 Otto Bock).

## PROSTHETIC KNEES

The prognosis whether the patient would be able to learn walking with an auto-adaptive knee joint was unknown because no comparable literature data was available. Therefore, a trial phase using C-legs was started, which contains an appropriated combination of safety and dynamics. The auto-adaptive knee joints allowed testing of the patients’ capabilities in using a free knee joint, while creating both a safe and dynamic ambulation condition. Additionally, during this process, the C-leg was the only microprocessor-controlled knee joint released for bilateral TFA with OPF.

The patient trained intensively with rapid progression. Regardless, the patient did not experience enough safety and stability with the C-leg while standing due to the continuous contraction of the hip extensors and lordosis of the lower back required to maintain control of the center of mass. In an attempt to solve the aforementioned issues, a trial period request with Genium knees was sent to the supplier. After an initial hesitation to release the Genium knee to a patient with a bilateral TFA in combination with an OPF, the supplier honored the request and the treatment was successfully continued.

## OUTCOMES

After 4 weeks of training with the Genium knees, the patient was able to walk short distances, stand up from the floor, walk on ramps, descend a stair or hill, and rise up from a chair. Due to a diminished balance and decreased muscle strength due to the short stump length, a standing position could be held using 1 crutch. The life habits questionnaire (LIFE-H)^14^ and the lower extremity functional scale (LEFS)^15^ was used to evaluate the disabilities of the patients’ daily life activities before and after the OPF.

**FIGURE 2:**
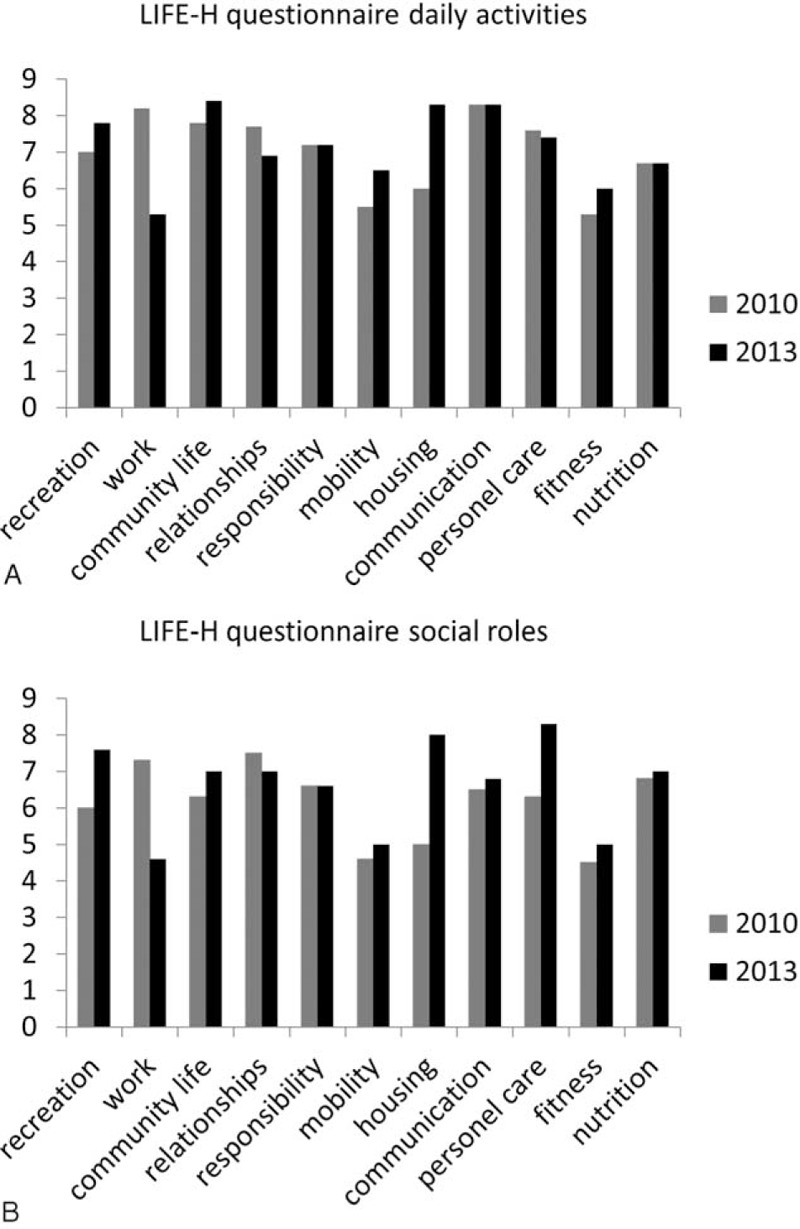
a LIFE-H questionnaire—domain daily activities. This questionnaire is divided in 12 categories (education was not applicable for this participant) and a total amount of 68 items. The maximum score is 9 points per category. The gray bar shows the outcome of 2010 (before the OPF), and the black bar shows the results of 2013 (post OPF). Figure LIFE-Habits (LIFE-H) questionnaire—domain social roles. This questionnaire is divided in 12 categories (education was not applicable for this participant) and a total amount of 68 items. The maximum score is 9 points per category. The gray bar shows the outcome of 2010 (before the OPF), and the black bar shows the results of 2013 (post OPF). LIFE-H = LIFE-Habits, OPF = osseointegrated prosthesis fixation.

The LIFE-H “domain daily activities” are higher in 2013 compared to 2010, when examining the categories recreation, community life, mobility, housing, and fitness (Figure 2a). The LIFE-H “domain social roles” are summarized in Figure 2b. The items recreation, community life, mobility, housing, communication, personal care, fitness, and nutrition were increased after 3 years. In contrast, the items work, interpersonal relationships, and personal care were decreased in 2013 compared to the results of 2010. The items responsibility and nutrition remained unchanged between 2010 and 2013.

**FIGURE 3:**
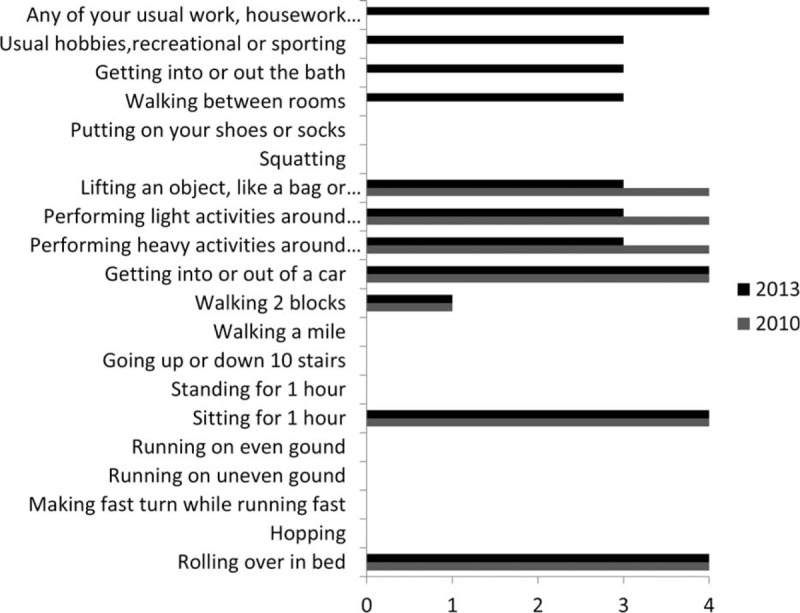
Lower extremity functional scale (LEFS). The gray bar shows the results of 2010, and the black bar shows the results of 2013. Scale varies from 0 to 4, 0 indicates extreme difficulties and 4 indicates no difficulty. LEFS = lower extremity functional scale.

The LEFS questionnaire (Figure 3*)* showed that the patient was capable of performing more activities in 2013 compared to 2010. Furthermore, the patient was able to walk two blocks, navigate between rooms, get in- and out of bath, performing the patient's regular hobbies, recreational and sport activities and the usual work activities, which was not possible in 2010. Activities, such as walking short distances, walking on ramps, rising up from a chair and working, were impossible in 2010 because of the inadequate fitted prosthesis.

## DISCUSSION

This case report demonstrates that OPF increased the mobility and the satisfaction in a bilateral TFA patient when compared to the previous situation without a prosthesis. OPF in combination with the Genium knees allowed the patient to perform more activities in daily life. However, some contradictory results are presented.

The condition “education” changed independently from the OPF, whereas other items, such as “satisfaction” and “personal care”, influenced the results negatively. The unexpected results may have stemmed from a change in the personal conditions of the patient, independent of the OPF, resulting in questions which were no longer applicable to the patient 3 years after the initial assessment. In 2010, the participant was used at a military unit, living with his parents and forming a robust social network with both his family and friends from the army. The social context changed in 2013, because the patient moved from his parents’ house to his own place, resulting in a greater independence from his family.

At the beginning of the rehabilitation process, the priority was neurological improvement, due to the many impairments. Four years after the initial accident, the patient had new goals and perspectives, which influenced the rehabilitation process.

The patients’ bilateral TFA is the main cause accountable for the limited score on the LEFS. Due to the inability to execute a squat, run, hop, or walk stairs because of lacking the required muscles to perform these activities. Walking a mile or standing for 1 hour requires a substantial amount of energy for the individual. Additionally, before undergoing the OPF procedure, the participant hoped to be able to execute the tasks that he was unable to perform, including light and heavy activities around home and lifting objects from the floor. After the OPF procedure, the patient admitted that he may have overestimated the expected capabilities.

As is demonstrated in the case report, 1 crucial benefit of a well-trained individual is strong muscles strength, as this is required to optimize the mobility of the hip and trunk.^16^ Not all individuals with bilateral TFA have this beneficial physical condition before the surgical procedures. Moreover, individuals with a short stump require more stability from the hip muscles and more mobility from the pelvis and trunk, compared to individuals walking with a prosthesis fixed to a larger stump.^17^ The decreased moment arms and muscle fiber lengths available to joints result in a greater effort for the individual to walk safely.

Furthermore, the cerebral trauma of the patient impacted the functional capacity of the right side of the body. The decreased functional capacity might have influenced the prosthetic device rehabilitation process by impacting the coordination of the right stump. However, the presence of the patients’ neurological disability was not described in detail in this case report.

The required rehabilitation period was extensive due to the involvement of two surgical procedures and the minimal time necessary to achieve the full body-weight load. An interval of three and a half years was necessary before the patient decided to undergo an OPF.

Subsequently, the rehabilitation treatment amounted to another year, where the patient learned to walk with the osseointegrated prosthesis. Studies on unilateral TFA with OPF show roughly the same duration of rehabilitation.^18–20^ Literature demonstrates that the rehabilitation process might lengthen, due to infections and co-morbidity present in given individuals.

The disadvantages of OPF procedure, including fractures and infections, have not completely been reported in the literature and need to be considered.^21,22^ The use of OPF has several advantages that make it a more suitable device in contrast to the common problems experienced with a socket-prosthesis. The osseous integration in OPF results in lower energy expenditure while walking compared to previous devices, such as a pelvic belt and separated sockets. Further, the range of motion of the hip is increased and the pelvic tilt is reduced, which improves the observed walking pattern.^16^

At the end of the rehabilitation program, the patient received 2 Genium knees, which have been proven to provide better control while walking on ramps and uneven surfaces, in comparison to C-legs.^23,24^ However, these studies are based on a unilateral Genium knee application. In the case of a bilateral application of Genium knees, some activities remain near to impossible to perform, including climbing stairs, rising out of a chair, and sitting down in a controlled manner. In our case, the patient did not have enough muscle strength to perform the above-mentioned motions. Then again, the use of the prosthesis after OPF might have disadvantages in daily life. In account of the direct anchorage of the implant to the residual bone, it is possible that falling could damage the intramedullary portion of the implant and the bone.^25^ Thus, a prerequisite in a candidate for OPF is sufficient balance and stability. On the long term, 1 study reported that the bone mineral density reduces and the bone strength decreases during use of an OPF.^26^

In conclusion, although several studies clearly demonstrate the benefits of using an OPF, studies describing the long-term effects are lacking.^21,27^ In this specific case we conclude that the quality of life improved through the use of an OPF. However, OPF might not be the appropriate device for every individual with TFA, due to varying bone compositions and co-morbidities.^28^
